# Alveolar Soft Part Sarcomas: Molecular Pathogenesis and Implications for Novel Targeted Therapies

**DOI:** 10.1155/2012/428789

**Published:** 2012-04-08

**Authors:** Bryan Mitton, Noah Federman

**Affiliations:** ^1^Division of Pediatric Hematology/Oncology, Department of Pediatrics, Mattel Children's Hospital at UCLA, UCLA David Geffen School of Medicine, 10833 Le Conte Avenue, Los Angeles, CA 90095-175, USA; ^2^UCLA Pediatric Bone and Soft Tissue Sarcoma Program, The UCLA Sarcoma Program, Nanotechnology Program Area, UCLA Jonsson Comprehensive Cancer Center, Los Angeles, CA 90095, USA

## Abstract

Alveolar soft part sarcoma (ASPS) is a very rare soft tissue sarcoma which arises primarily in children and young adults. Despite its unique histology and well-characterized genetic translocation, many questions remain regarding the pathogenesis and treatment of this tumor type. Though collective clinical experience with this tumor type spans more than 60 years, there has been little progress made in treating this uncommon but frequently fatal disease. This paper focuses on the available data regarding its molecular pathogenesis and insights into targeted therapeutics as well as the results of clinical trials performed to date to hopefully improve the outcome of patients with this rare malignancy.

## 1. Introduction

Alveolar soft part sarcoma (ASPS) is a very rare sarcoma which arises primarily in children and young adults. Despite more than 60 years of experience with ASPS, several fundamental questions regarding this tumor type remain unanswered. The tissue of origin for ASPS remains unclear; the risk factors which lead to tumorigenesis and clinical progression are unknown, and the optimal approach to therapy is undefined. Though significant progress has been made in the molecular characterization of this tumor in the past 10 years and a number of exciting clinical trials are underway, this tumor has eluded elementary characterization for many decades.

## 2. Clinical Features of Alveolar Soft Part Sarcomas

Alveolar soft part sarcoma (ASPS) accounts for approximately 0.5–1% of all soft tissue sarcomas [1]. It is diagnosed most commonly in those between 15 and 35 years of age; in some large case series, the incidence is slightly increased in young females by a ratio of 3 : 2 compared to age-matched males [2]. Disease usually presents as a painless, soft, slow-growing lesion that rarely causes functional impairment. In children, ASPS most frequently occurs in the head and neck region, especially the tongue or orbit; in older adults, it arises from muscles of the lower or upper extremities [3–5].

Typically, this tumor grows indolently for years. Metastasis is detected in ~20% of patients at diagnosis and develops in ~80% of patients during the course of treatment [2]. Risk factors for developing this tumor remain undefined, but the risk for metastatic disease includes older age and larger tumor size (>5 cm) at diagnosis [2, 6]. ASPS, as with most other sarcomas, most often metastasizes to the lungs, but central nervous system involvement is also frequently described; indeed, ASPS has been reported to metastasize to the brain more frequently than any other form of high-grade sarcoma [1, 7–9]. Though there have been no cases reported of brain metastasis in the absence of lung metastasis, liver metastasis and intraosseous extension of the tumor without widespread disease have been described. The primary tumors are often large, with a mean size of 6.5 cm in one study, and typically high vascular, such that they sometimes present as a pulsatile mass [2]. On magnetic resonance imaging, they may appear similar to arteriovenous malformations [10]. Irregular intravascular extension is present at the tumor margins in almost all cases. The 5-year overall survival rates range from 45 to 88%, with a 20-year survival of approximately 15%; the median survival time is 6 years. Survival is dictated largely by disease stage and the size of the primary tumor [1–3, 6].

## 3. Histologic Features of Alveolar Soft Part Sarcomas

Christopherson et al. were the first to designate these tumors as “alveolar soft part sarcomas” in 1952, given their unique histologic appearance and uncertain tissue origin [11]. To date, the definitive origin of this tumor remains unknown. There is some immunohistochemical evidence suggesting that ASPS may arise from striated muscle or pericytes, this remains controversial [12–15]. Primary ASPS tumor sites have also been reported in tissues where skeletal muscle is absent, such as in the stomach, breast tissue, and the female genital tract [16–18].

ASPS tumors are histologically distinctive. Interestingly, this tumor type was originally named for its striking architectural similarity to respiratory alveoli; classically, poorly differentiated tumor cells are arranged in nests separated by thin layers of connective tissue containing sinusoidal vascular channels, which in turn are lined by thin endothelium [3]. A histologic variant of ASPS has been described in young patients with lingual ASPS, which lacks the typical cellular discohesion and thus has a solid “nonalveolar” growth pattern [19]. Smetana and Scott in 1951 were the first to describe the hallmark intracytoplasmic crystals of ASPS [20]. These crystals are rod shaped, coarse, and basophilic bodies of unknown significance, though they have been shown to contain monocarboxylate transporter 1 and CD147 [21]. These cells demonstrate PAS-positive granules in almost all tumors and often stain positively for desmin [4]. Electron microscopy demonstrates rhomboid, rod shaped crystals consisting of rigid fibrils. In spite of these features, ASPS still may present a diagnostic challenge, as it may resemble metastatic renal cell carcinoma, paragangliomas, granular cell tumors, or melanomas [3]. Preoperative imaging, usually with magnetic resonance imaging, is the standard of care. Core needle biopsy or fine needle aspiration should be considered before definitive surgery. Because of the presence of intracellular crystals, fine-needle cytology can often offer sufficient material for diagnosis, but as with any diagnosis of solid tumor, excisional biopsy may be required to diagnose this rare tumor.

We now turn our attention to the available data concerning the pathogenesis of this unique tumor, as well as the therapeutic strategies now available.

## 4. Molecular Pathogenesis of Alveolar Soft Part Sarcoma

ASPS is characterized by an unbalanced translocation between the X chromosome and chromosome 17, first described in a seminal paper by Ladanyi et al. in 2001 [22]. The der(17)t(X;17)(p11;25) translocation is found in all ASPS tumors studied; in the majority of ASPS tumors, this translocation is found in an unbalanced form, resulting in loss of heterozygosity at 11q25 [23]. Interestingly, this translocation is also found in a distinctive subset of renal cell carcinomas which frequently have papillary architecture, usually in the balanced form [24].

**Figure 1:**
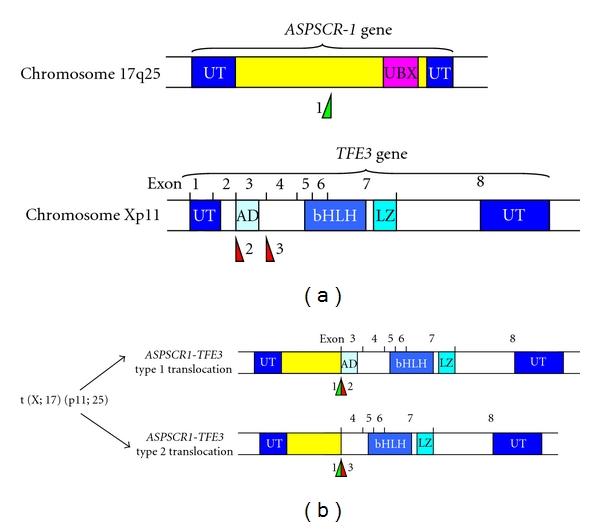
The t(X;17)(p11;25) translocation. (a) The ASPSCR-1 gene is located at chromosome 17q25 and the TFE3 gene at Xp11. The breakpoint found in the ASPSCR-1 gene is marked at “1”, and the two defined breakpoints in the TFE3 gene are marked “2” and “3”. (b) Following translocation, two variants of the ASPSCR-1-TFE3 fusion gene can be created. The Type 1 translocation retains the N-terminal activation domain of the TFE3 gene.

Elegant studies defined the precise base pair position at which this translocation occurs; the resultant fusion protein involves the Alveolar Soft Part Sarcoma Critical Region-1 gene (*ASPSCR-1*) located on chromosome 17q25 and the Transcription Factor for Immunoglobulin Heavy-Chain Enhancer 3 (*TFE3*) gene, located on chromosome Xp11.22 [22]. Structurally, the N-terminus of the *ASPSCR-1* gene is fused in-frame with the *TFE3* (Transcription Factor E3) gene at exon 3 (Type I) or exon 4 (Type 2), resulting in one of two novel, functional *ASPSCR1-TFE3* fusion proteins which are capable of inducing aberrant transcription of *TFE3*-regulated genes (Figure 1). At the molecular level, the first 234 aminoterminal aminoacids from *ASPSCR-1* are fused to the *TFE3* gene at aminoacid positions 280 or 315. There are no data as to whether these two different fusion products result in clinically different diseases in terms of presentation, metastasis, or prognosis.

The *TFE3* gene is a member of the microphthalmia transcription factor/transcription factor E (MITF-TFE) family of basic helix-loop-helix leucine zipper (bHLH-Zip) transcription factors along with *MITF*, *TFEB,* and *TFEC* [25]. The TFE3 gene expresses the TEF3 (Transcription Enhancing Factor 3) protein. The MITF-TFE family of transcription factors functions as homo- or heterodimers. They are ubiquitously expressed, and each of the possible binding pairs have been demonstrated *in vitro*; it has been suggested the specific gene program activated by the TEF3 protein depends mainly on its binding partners, which is dynamic and tissue specific [26]. The TEF3 protein, encoded by the *TFE3* gene, also interacts with transcriptional regulators such as E2F3, SMAD3, and LEF-1, and plays a variety of roles in cell growth and proliferation [27].

A number of clues suggest the potential of the *TFE3* gene as a protooncogene. Early evidence demonstrated that the TEF3 protein activates transcription via binding of its E3 motif to the EBox DNA consensus sequence (CANNTG) in the immunoglobulin heavy-chain enhancer [28]. TEF3 regulates a number of metabolic genes which possess the EBox in their promoters, such as the S-phase regulator cyclin E, in an E2F3-dependent manner [27]. Interestingly, TEF3 may confer resistance to cell cycle arrest signals and can override arrest when ectopically expressed. For example, the presence of TEF3 can override Rb-induced cell cycle arrest, and can block the antimitogenic effects of TGF-*β* in mammalian cells [27]. TEF3 has an activating domain at both the N- and C-termini; *in vitro* deletion of the N-terminal domain results in a dominant negative form of the factor that interferes with the function of the full-length protein [29]. This activation domain is lost in the Type 1 *ASPSCR1-TFE3* gene translocation and not the Type 2 variant, though there are no clear phenotypic differences in the tumors that arise from each of these translocations. Interestingly, 15% of cases of renal cell carcinomas in which TFE3 gene fusions are detected is associated with prior exposure to chemotherapy [30]. A strong association between prior chemotherapy and the subsequent development of ASPS has not been demonstrated.

The *ASPSCR-1* gene has been alternatively termed in the literature *TUG* (Tether-containing UBX domain for GLUT4), *ASPL* (alveolar soft part sarcoma locus), *UBXN9*, *UBXD9*, and *FLJ45380*. This protein is expressed ubiquitously, though it has highest expression in the adult heart and skeletal muscle [22]. For a number of years following the discovery of the *ASPSCR1-TFE3* translocation, the function of the *ASPSCR-1* gene product was largely unknown; there are now data that show that it functions as a tether which interacts with the glucose transporter type 4 (GLUT4) and cellular/organellar membranes [31]. The ASPSCR-1 protein appears to sequester the GLUT4 in intracellular vesicles in muscle and adipocytes in the absence of insulin and facilitates redistribution of this channel to the plasma membrane following insulin stimulation. In the context of a novel fusion protein, it is unclear how the anchoring functionality of ASPSCR-1 may influence the function of TEF3.

One may speculate that the novel N-terminus of the *ASPSCR1-TFE3* fusion protein may interfere with or obviate the normal activation or dimerization functions of TEF3 to the extent that normal transcription is deranged. TEF3 may bind an alternative transcription factor, leading to aberrant transcriptional programs or simply homodimerize in the absence of an activating signal and remain constitutively active. The specific role of an N-terminal segment of the TUG protein is unclear, though hypotheses could be made that the presence of this peptide alters dimerization or activation of the TEF3 peptide component. It is important to note, however, that the *TFE3* gene is associated with other tumors and a number of oncogenic translocations. The t(X;17)(p11;25) translocation is additionally detected in some cases of perivascular epithelioid cell neoplasms (PEComas), and as mentioned above, and also is found in papillary renal cell adenocarcinomas, more frequently in the pediatric population [32]. Within this subset of renal cell adenocarcinomas, four other *TFE3* gene translocations have been described, as shown Table 1 [22, 33–39]. Additionally, novel chromosomal translocations have been identified which await definition of the involved gene loci. Thus, five discrete translocations associated with oncogenesis have been identified to date, and these translocants are thought to serve diverse functions. This suggests that perhaps the loss of the native N-terminus of the *TFE3* gene is more important in tumorigenesis than the particular composition of the ectopic genetic material added to it.

**Figure 2:**
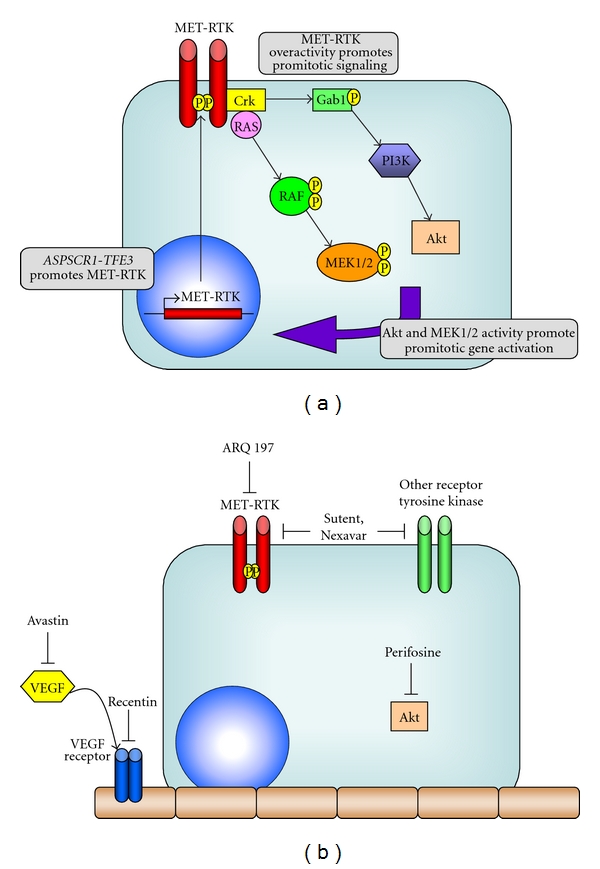
Signaling schematic for ASPS tumors and novel therapeutic targets. (a) Overexpression of the MET tyrosine receptor kinase is induced by the ASPSCR1-TFE3 fusion protein. This leads to intracellular activation of the promitotic growth kinases Akt and MEK1/2; these in turn lead to unchecked cellular proliferation. (b) Targets for novel therapeutics include the VEGF tyrosine kinase receptor and its ligand (Recentin and Avastin, resp.), as well as tumor cell receptor tyrosine kinases (Sutent, Nexavar, and ARQ 197). Perifosine is a unique inhibitor of the Akt kinase.

In the last few years, large strides have been made in ascertaining how the unique ASPSCR-1-TEF3 fusion protein leads to tumorigenesis. Tsuda et al. (2007) identified that the ASPL-TFE3 fusion protein induces strong overexpression of the MET receptor tyrosine kinase gene in ASPS cells [40]. This group showed that in the presence of its ligand, hepatocyte growth factor (HGF), the MET receptor tyrosine kinase underwent strong autophosphorylation, activating robust downstream signaling of the MAP kinase and PI3K/Akt pathways (Figure 2). Inhibiting expression of MET by RNA interference or a specific inhibitor abolished the HGF-dependent MET activation, leading to decreased cell growth. These data provide a mechanism, whereby the presence of the ASPSCR1-TFE3 fusion protein could potentially induce cell mitosis. Interestingly, the *PSF-TFE3* and *NonO-TFE3* fusion proteins also activated this promoter, again implicating TEF3 as the primary determinant of this phenomenon. As mentioned, TEF3 may have broad roles in regulating mitosis and the release of cell cycle blockade; additional parallel signaling circuits may be similarly activated. Nonetheless, the induction of the MET receptor tyrosine kinase pathway by the *ASPSCR-1-TFE3* fusion protein represents a major advance in our understanding of this tumor.

## 5. Contemporary Therapeutic Approaches

The majority of clinical data concerning the outcomes for those diagnosed with ASPS comes from large case series spanning many decades, given the rarity of this tumor. Lieberman et al. [2] provide the largest descriptive study of patients with ASPS to date; data from 102 patients with ASPS were collected from the years 1923 to 1986, and their outcomes are studied. Aggregate 5-year survival was 62% at 5 years and 18% at 20 years (median 7 years). Of 69 patients without metastasis at diagnosis, 60% remained free of metastasis after 5 years and 15% after 20 years (median 6 years). Those who developed metastases following diagnosis had a median survival of 2 years after discovery. The most common sites for metastasis noted were lung, bone, and brain, though it was noted that brain metastasis was never detected in the absence of lung metastasis. Of those with metastasis at diagnosis, the median survival was 3 years.

This case series provided the first evidence that surgical intervention appears to be the only clinically effective therapy for the treatment of ASPS. Indeed, adjuvant therapies including chemotherapy or radiation therapy yielded no significant difference in overall patient survival regardless of stage, nor in the context of the presence, absence, or later development of metastasis. In this study, of 91 patients with localized disease who underwent primary surgical excision, 18 patients were also treated with radiotherapy, 2 with chemotherapy, and 2 patients with both modalities. The rate of local recurrence was not significantly different compared to those treated with no adjuvant therapy. Similarly, adjuvant therapy yielded no significant difference in the number of patients who went on to develop metastases. Finally, no treatment modality, including chemotherapy, radiation therapy, or surgical resection appeared to provide a survival benefit compared to those who were not treated after the development of metastasis.

A number of similar conclusions were drawn from a more recent study. Portera et al. [1] report data from 74 patients collected over almost 40 years; in this study, 65% presented with American Joint Committee on Cancer (AJCC) stage IV (metastatic) disease and the rest with AJCC Stage II or III disease. In this series, those with nonmetastatic disease were treated with surgery alone (9 patients) or surgery plus external beam radiotherapy (10 patients). Three patients were also given neoadjuvant doxorubicin prior to surgical resection. For those with localized ASPS, the 5-year local recurrence free, distant recurrence free, disease free, and overall survival rates were 88%, 84%, 71%, and 87%, respectively. Over a decade, 2 of 22 patients with localized disease developed local recurrence, and 3 developed lung metastasis, reflecting percentages similar to those reported by Lieberman et al. From these data, radiation therapy again did not appear to greatly influence survival or the development of metastases, though low patient numbers preclude definitive conclusions.

Of 48 patients presenting with Stage IV disease in this study, 73% had metastasis to one organ, which was the lung in ~90% of cases. In those with more than one site of metastasis, the lung was always involved, and brain metastases were found in 9 of 29 patients. Twenty-six patients of 33 with Stage IV disease were given systemic chemotherapy which included vincristine and/or cyclophosphamide (prior to 1970) or doxorubicin-based therapy. The majority of patients treated with chemotherapy (58%) developed disease progression. This population median survival was 40 months, with a 5-year survival rate of 20%. These data again mirror previously described data.

Importantly, this case series showed that with more “modern” chemotherapy regimens utilizing vincristine, cyclophosphamide, or doxorubicin, clinical response was disappointing. Among the 26 patients with Stage IV disease who received chemotherapy and the 3 patients with localized disease who received neoadjuvant doxorubicin-based systemic therapy prior to resection, only a single patient stage IV responded, though there was a complete response. Chemotherapy yielded no minor or incomplete responses. Thus, this study provided little evidence that contemporary systemic chemotherapy elicits a survival benefit.

Finally, Kayton et al. [6] describe data from 20 patients collected over 30 years. These patients ranged in age from 6 to 25, with 35% of patients presenting with Intergroup Rhabdomyosarcoma Study stage IV (metastatic) disease. Patients with IRS Stage I (local) disease underwent surgery alone, and none had evidence of local recurrence at followup, which ranged from 4 to 290 months; 20% of patients, however, developed detectable metastases at followup. For those with IRS Stage IV disease, a variety of approaches including radiation therapy to the primary tumor or metastases, chemotherapy, and excision of the primary mass ± metastasectomy were attempted. The authors note no partial or complete responses to a wide variety of chemotherapy regimens attempted, including antimetabolites, alkylating agents, mitotic inhibitors, anthracyclines, or biologic agents. Similarly, small numbers precluded clear conclusions regarding radiation therapy. The aggregate 5-year survival for all patients with ASPS was 83%, with 5-year progression-free survival of 22%; of note, those presenting with a primary site tumor >5 cm in size all died before 5-year followup, whereas those with tumor size <5 cm had a ~70% progression-free survival. The data from this case series emphasize the importance of complete microscopic resection with negative margins in those with early stage disease, as well as the large bearing tumor burden plays in determining survival. Furthermore, for patients who present with metastases, the authors note very short progression-free survival (median 12.5 months) but much longer time of overall followup (median 36 months). This may reflect the indolent nature of the disease, rather than the effectiveness of surgical resection per se.

From these reports, there are no data to support the use of any modality of therapy aside from surgery for ASPS; no significant survival advantages have been achieved by utilizing chemotherapy or radiation for patients who have local or metastatic disease at the time of diagnosis as compared to patients who are not treated. The authors of these studies, however, note the importance of palliation, when necessary, and that though there are no data demonstrating survival benefit, radiation therapy should be considered in specific cases where there is a large primary tumor burden. Our recommendations based on these data are expectant observation when metastatic disease is low volume and nonprogressive on serial imaging.

## 6. Targeted Therapies and Clinical Trials

The resistance of ASPS tumors to conventional chemotherapies and radiation makes this type of tumor challenging to treat; however, a number of exciting clinical trials are underway which are investigating novel targeted therapies. These newer agents offer many advantages over traditional chemotherapeutic agents and radiotherapy, such as reduced toxicity and daily outpatient use. The theoretical ability to indefinitely continue minimally toxic therapy becomes especially important, given the indolent nature of ASPS even in the context of metastatic disease. A number of trials underway currently seek to focus on the overactivity of the MET receptor tyrosine kinase gene induced by the ASPSCR1-TFE3 fusion protein. In addition, the vascular nature of this tumor also suggests a potential role for antiangiogenic agents.

One such trial focused on ARQ-197 (ArQule), a selective inhibitor of the c-Met receptor tyrosine kinase. This drug was tested in a Phase II study (NCI Clinical Trial NCT00557609) examining the drug's effect on Microphthalmia Transcription Factor Family (MiT) tumors, which include ASPS, clear cell carcinoma, and renal cell carcinomas bearing a TFE3 translocation [41]. Preliminary data presented at the 2009 American Society of Clinical Oncology indicated that 15 of 17 patients treated with ARQ-197 demonstrated stable disease at ~29 weeks of therapy, with a disease control rate of ~80% [42]. This drug has an excellent safety trial following the completion of three Phase I trials, and although data are not mature, they appear promising.

Along these same lines, multitargeted tyrosine kinase inhibitors have also been investigated in small trials; sunitinib malate (SU11248, Sutent, Pfizer), a multitargeted RTK inhibitor with antiangiogenic properties approved for treatment of GIST and renal cancer, was recently trialed in 8 patients [43]. Five patients showed partial response, one had stable disease and one progressed. Similarly, a phase II trial with sorafenib (Nexavar, Bayer, NCI Trial NCT00330421), another multitargeted tyrosine kinase inhibitor, showed that in 28 patients, 12 had partial response and 6 had stable disease, for a disease control rate of 78% [44]. Other clinical trials underway for the treatment of ASPS include the Akt inhibitor, KRX-0401 (Perifosine, Keryx/AOI, NCI Clinical Trial NCT00401388), and antiangiogenic approaches such as bevacizumab (Avastin, Genentech) or Cediranib (Recentin, AstraZeneca) [45–47]. Preliminary data from two phase II trials using Cediranib (Study code 2171IL0038) also presented at the 2009 American Society of Clinical Oncology showed that four of seven patients had a partial response, two patients had a confirmed reduction in tumor size, and one patient demonstrated stable disease [48]. Further clinical trials for Cediranib are currently open to accrual (NCI Clinical Trials NCT01337401 and NCT00942877) [49, 50].

## 7. Future Directions

The discovery of further novel therapeutic targets has been aided by a number of recent reports providing broad and targeted gene expression and immunohistochemical arrays. Together, Stockwin et al. and Lazar et al. provide comprehensive studies characterizing gene expression profiles of ASPS, with a focus on a number of key players involved in angiogenesis, cell proliferation, and metastasis [51, 52]. For instance, these manuscripts both identified upregulation of MDK (midkine or neurite growth-promoting factor-2) and Jag-1 (Jagged-1), which are regulators of angiogenesis. MDK is a low molecular weight growth factor which antagonizes VEGF signaling and that appears to be upregulated in multiple solid tumors; Jag-1, in contrast, is the ligand for the Notch-1 receptor and is a potent proangiogenic signal [53–56]. There are numerous suggestions that both these molecules represent excellent targets for novel therapeutics, and there are preclinical data that are promising [57–59]. Stockwin et al. also demonstrated the expression of GPNMB, a transmembrane protein which bears homology to the melanoma antigen pMEL17. CDX-011, or Glembatumumab (Celldex), is an antibody targeted against GPNMB (Transmembrane Protein NMB); this antibody was conjugated to vedotin (monomethyl auristatin E), a highly potent antimitotic agent, in recent Phase 2 trials for advanced breast cancer and late-stage melanoma [60, 61]. These data provide a rational basis for use of this drug in ASPS. Finally, Martignoni et al. demonstrate that all tested samples of alveolar soft part sarcomas diffusely express cathepsin K, whose expression is driven by MITF in osteoclasts; interestingly, renal cell carcinomas with the same *ASPSCR1-TFE3* translocation do not detectably express this protease [62]. Argani et al. also reported the expression of cathepsin K in PEComas [32]. Odanacatib (MK-0822, Merck) is a monoclonal antibody against cathepsin K and has been studied in women with breast cancer with bony metastases; again, cathepsin K may represent a potential therapeutic target [63].

## 8. Conclusions

In summary, alveolar soft part sarcomas are rare, unique malignancies which grow indolently and remain difficult to treat despite decades of clinical experience. Recent data have linked the specific t(X;17)(p11;25) translocation found in all ASPS tumors studied to the overexpression of the promitotic MET receptor tyrosine kinase, providing a model for tumorigenesis. At this point, surgical methods are the most efficacious means of disease treatment; there are no convincing data in support of conventional chemotherapy or radiation therapy. New molecular therapies targeted to tyrosine receptor kinases and antiangiogenic agents have yielded promising data thus far, and these next-generation therapies may soon comprise first-line treatment for this tumor type.

## Figures and Tables

**Table 1:** Translocations involving the *TFE3* gene.

Fusion protein	Genetic translocation	Function of gene N-terminal to TFE3	Reference
*TFE-ASPSCR-1 *	t(X;17)(p11;25)	Tether-containing UBX domain for GLUT4 (*ASPSCR-1*)	Ladanyi et al. [22, 24]
*TFE-PRCC *	t(X;1)(p11.2;q21.2)	Papillary renal cell carcinoma translocation-associated (*PRCC)* gene	Weterman et al. [33] and Sidhar et al. [34]
*TFE-PSF*	t(X;1)(p11.2;p34)	PTB-associated splicing factor (*PSF*)	Clark et al. [35]
*TFE-NonO*	inv(X)(p11.2;q12)	Non-POU-domain-containing, octamer-binding (*NonO*) gene	Clark et al. [35]
*TFE-CLTC*	t(X;17)(p11.2;q23)	Clathrin heavy-chain (*CLTC*) gene	Argani et al. [36]
Unknown	t(X;10)(p11.2;q23)	Unknown	Dijkhuizen et al. [37]
Unknown	t(X;3)(p11;q23)	Unknown	Argani et al. [38]
Unknown	t(X;19)(p11.2;q13.1)	Unknown	Armah et al. [39]
